# 3.0T Cardiac Magnetic Resonance Quantification of Myocardial Extracellular Volume using different delay time of post-contrast T1 mapping for the Diagnosis of Cardiac Amyloidosis

**DOI:** 10.1186/1532-429X-18-S1-P316

**Published:** 2016-01-27

**Authors:** Lu Lin, Yining Wang, Jian Cao, Lingyan Kong, Jing An, Tianjing Zhang, Bruce S Spottiswoode

**Affiliations:** 1 Radiology, Peking Union Medical College Hospital, Beijing, China; 2 Siemens Shenzhen Magnetic Resonance Ltd., beijing, China., Beijing, China; 3 MR Research and Development, Siemens Healthcare, Chigago, IL USA

## Background

Cardiac involvement in systemic light-chain (AL) amyloidosis is caused by the extracellular deposition of misfolded AL immunoglobulin and carries a poor prognosis. Cardiac magnetic resonance (CMR) T1 mapping and extracellular volume (ECV) measurements may have advantages over late gadolinium enhancement (LGE) for early diagnosis and quantifying of amyloid burden. The aim of this study was to explore the diagnostic value of ECV for cardiac amyloidosis and compared ECV values calculated by post-contrast T1 mapping got in different delay time.

## Methods

The study received local ethical approval and all patients gave written informed consent. A consecutive series of patients referred to clinical CMR exam were included. The amyloidosis group included patients diagnosed of systemic AL amyloidosis with definite clinical cardiac involvement. The control group included other patients with neither clinical evidence for infiltrative cardiomyopathy nor late gadolinium enhancement (LGE) found in CMR. All the patients underwent a standardized bolus contrast-enhanced CMR (3.0T) imaging composed of native, 5 minutes (min) and 15-20 min post-contrast T1 mapping using a Modified Look Lockers Inversion (MOLLI) recovery sequence in identical imaging locations, including a long axis 4-chamber (4ch) slice and a middle short axis 2-chamber (2ch) slice. ECV analyses using the 5 min (ECV1) and 15-20 min (ECV2) post-contrast T1 mapping images were accomplished semi-automatically by a dedicated software. Native T1, ECV1 and ECV2 value of identical region of interest in interventricular septum were measured and compared between amyloidosis group and control group by Mann-Whitney test. Paired ECV1 and ECV2 values in same region were compared by Wilcoxon test.

## Results

The amyloidosis group recruited 25 patients (mean age 54 ± 10 years, 13 men).10(40%) of them had an endomyocardium biopsy, all of which obtained a histological proof of cardiac amyloidosis by Congo red histology. The control group included 17 patients (mean age 41 ± 12 years, 12 men). Myocardial native T1, ECV1 and ECV2 all significantly elevated in amyloidosis group compared to control group both in 4ch slice ( 1475 ± 122 ms vs. 1285 ± 41 ms,0.51 ± 0.08 vs. 0.27 ± 0.03, 0.49 ± 0.08 vs. 0.27 ± 0.03, p = 0.000) and 2ch slice ( 1464 ± 114 ms vs. 1267 ± 40 ms, 0.54 ± 0.10 vs. 0.26 ± 0.03, 0.51 ± 0.09 vs. 0.26 ± 0.03, p = 0.000). In amyloidosis group, ECV1 was slightly higher than ECV2 both in 4ch slice (0.51 ± 0.08 vs. 0.49 ± 0.08, p = 0.013) and 2ch slice(0.54 ± 0.10 vs. 0.51 ± 0.09, p = 0.002), while in control groups ECV1 and ECV2 showed no significant difference in 4ch slice (0.27 ± 0.03 vs. 0.27 ± 0.03, p = 0.468) and 2ch slice(0.26 ± 0.03 vs. 0.26 ± 0.03, p = 0.925).

## Conclusions

Myocardial native T1, ECV calculated by 5 min and 15-20 min post-contrast bolus T1 mapping all significantly elevated in cardiac amyloidosis patients in 3T CMR. ECV calculated by 5 min post-contrast T1 mapping showed similar promising diagnosis potential for cardiac amyloidosis compared with 15-20 min post-contrast T1 mapping.

**Figure 1:**
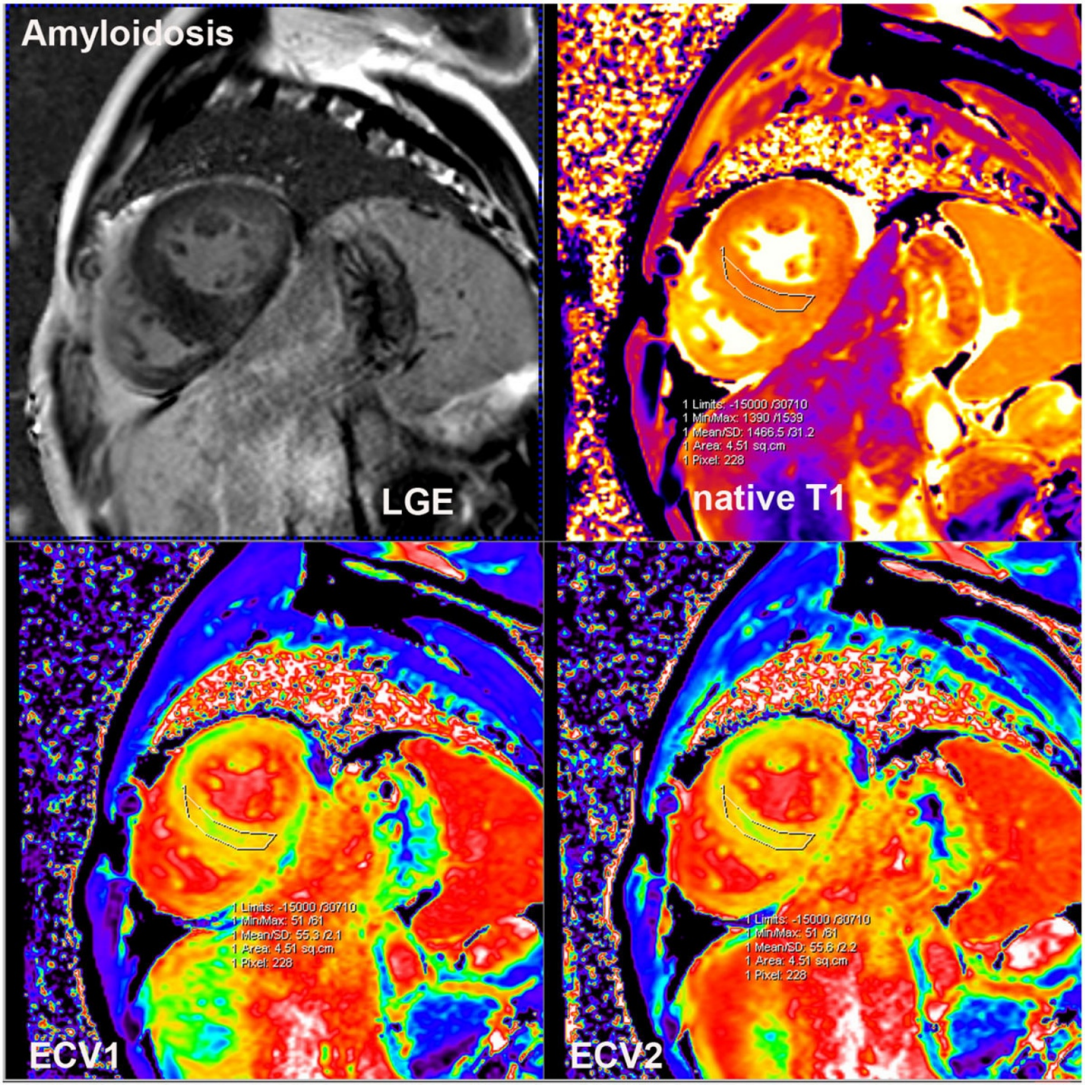
**LGE, native T1 mapping, ECV1(calculated by native and 5 min post-contrast T1 mapping) and ECV2 map(calculated by native and 15-20 min post-contrast T1 mapping) images in identical short axis 2 chamber slice of a endomyocardial biopsy proved cardiac amyloidosis patients**. Interventricular septum late enhancement was uncertain in LGE image, but region of interest measurements showed significantly elevated native T1(1466 ms), ECV1(0.55) and ECV2(0.55) of myocardium.

**Figure 2:**
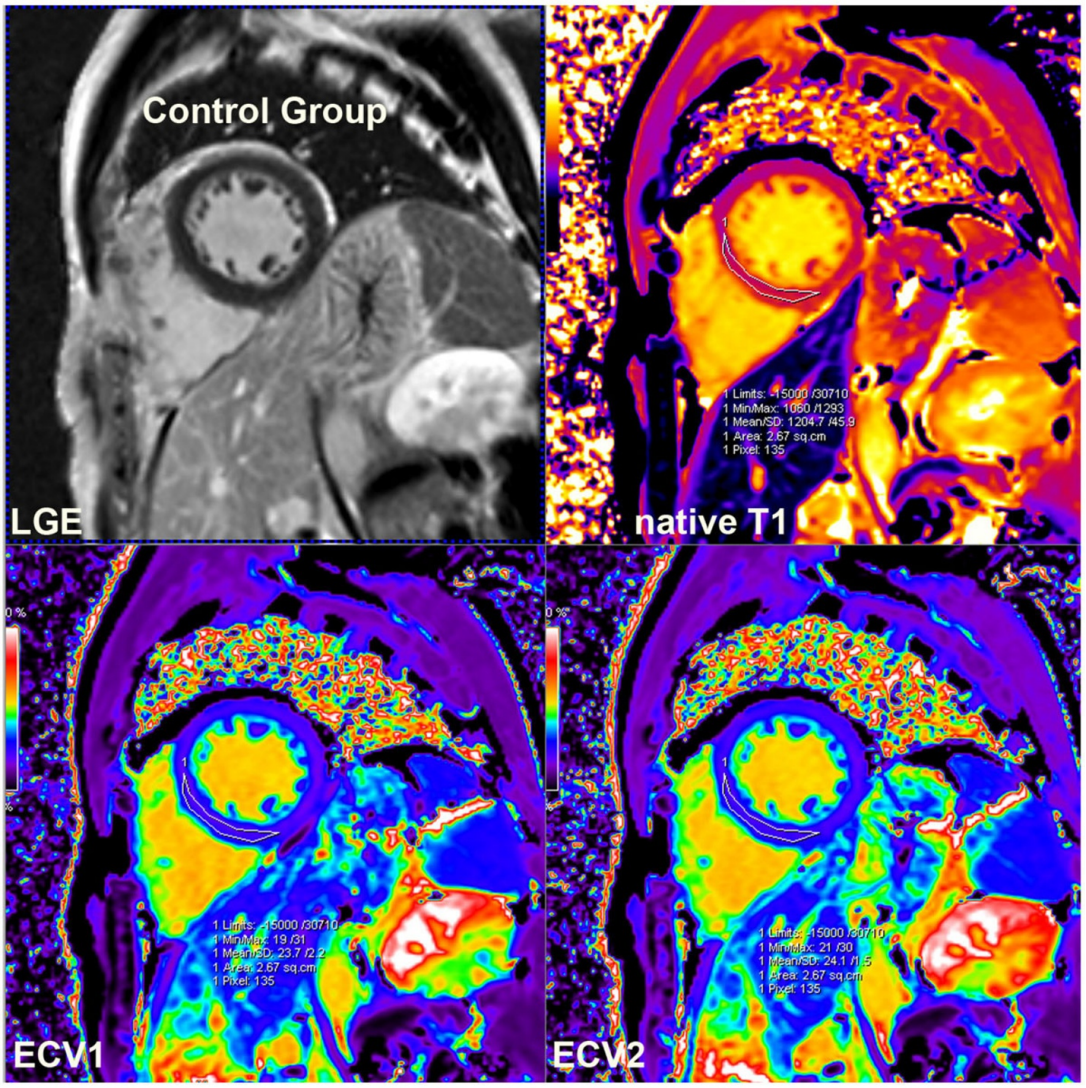
**LGE, native T1 mapping, ECV1(calculated by native and 5 min post-contrast T1 mapping) and ECV2 map(calculated by native and 15-20 min post-contrast T1 mapping) images in identical short axis 2 chamber slice of a control goup patients**. There was no prominent myocardial late enhancement.Interventricular septum region of interest measurements showed a relatively low native T1(1204 ms) and similarly normal ECV1(0.237) and ECV2(0.241).

